# A comprehensive atlas of endogenous peptides in maize

**DOI:** 10.1002/imt2.247

**Published:** 2024-11-11

**Authors:** Usman Ali, Lei Tian, Ruihong Tang, Shunxi Wang, Weiwei Luo, Shanshan Liu, Jinghua Zhang, Liuji Wu

**Affiliations:** ^1^ National Key Laboratory of Wheat and Maize Crop Science, College of Agronomy Henan Agricultural University Zhengzhou China

## Abstract

In this study, we present a comprehensive peptidomic atlas of 13 maize tissues, covering both vegetative and reproductive phases. Using a three‐frame translation of canonical coding sequences, we identified 6100 nonredundant endogenous peptides, significantly expanding the known plant peptide repertoire. By integrating peptidomic coexpression profiles with previously reported proteomic profiles, we found that the peptide abundance did not consistently correlate with the abundance of their source proteins, suggesting the presence of complex regulatory mechanisms. This integrated peptidomic and proteomic map can serve as a valuable resource for exploring the functional roles of endogenous peptides in maize development and facilitates the investigation of the functional relationship among genes, peptides, and proteins across various biological contexts.

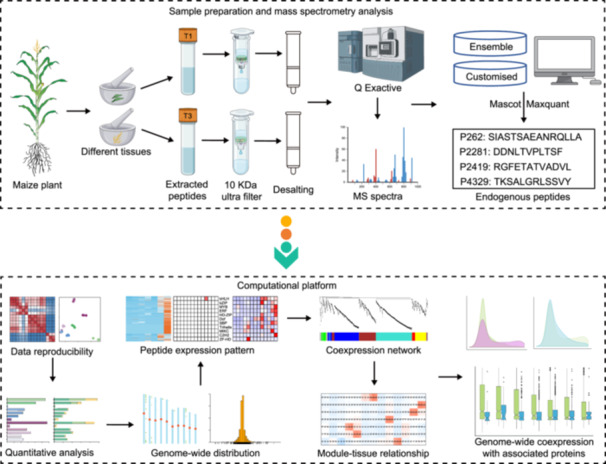

Endogenous peptides, typically consisting of 2–100 amino acid residues, are increasingly recognized as vital regulators of plant growth, development, and stress responses [1]. These small biomolecules play vital roles in various biological processes, including cell signaling, growth modulation, and defense mechanisms [2, 3, 4]. However, the majority of the research on endogenous peptides in plants has so far been limited to individual peptides or small groups of peptides, often focusing on their roles in specific biological processes. For instance, RAPID ALKALINIZATION FACTORs are known to maintain cell wall integrity during pollen tube and root growth in *Arabidopsis* [5, 6]. The peptide hormones such as Casparian strip integrity factor 1 (CIF1), CIF2, and C‐TERMINALLY ENCODED PEPTIDE participate in Casparian strip formation and root growth modulation, respectively [7, 8]. Despite these insights, a comprehensive investigation of endogenous peptides at genome‐wide level in plants has yet to be undertaken.

Peptidomics, the large‐scale study of endogenous peptides in plants, has lagged behind other molecular components, such as RNA and proteins. Currently, most genome‐wide studies in plants have focused on RNA and protein expression, particularly through transcriptomics and proteomics [9, 10, 11]. These studies have provided valuable insights into the roles of these molecules in various physiological processes, revealing complex regulatory networks underlying plant development. For example, transcriptome and proteome‐wide studies have revealed highly coordinated regulatory networks associated with flower development in *Arabidopsis* [10], fruit development in wild strawberry [12], and endosperm and mature leaf development in maize [9, 13]. However, while transcriptomics and proteomics have advanced our understanding of gene and protein regulation, endogenous peptides, generated from protein precursors or encoded by genes, have not been comprehensively analyzed on a genome‐wide scale in plants.

## RESULTS AND DISCUSSION

### Tissue atlas and overview of maize endogenous peptides

**Figure 1:**
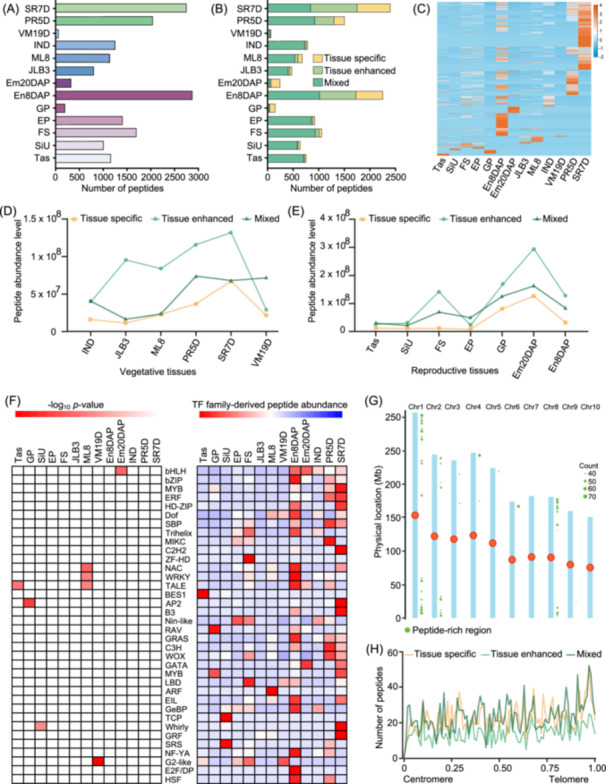
Quantitative analysis, abundance pattern, and chromosomal distribution of maize endogenous peptides. (A) Number of endogenous peptides identified in each tissue of maize. The tissue samples were taken from three individual plants and treated as biological replicates. (B) Expression specificity categories of peptides in each maize tissue. (C) Heatmap of total peptide abundance levels in each maize tissue. (D) The abundance pattern of tissue‐specific, tissue‐enhanced, and mixed peptides during the vegetative phase of the maize plant. (E) Abundance pattern of tissue‐specific, tissue‐enhanced, and mixed peptides during the reproductive phase of maize plant. (F) Familywise analysis of the abundance (expression) levels of peptides derived from transcription factors (TF). For each TF‐family‐derived peptide, the overenrichment of family members was determined for each tissue (left). The abundance of each TF‐derived peptide for a given TF family was summed for each tissue, followed by hierarchical clustering and row normalization (right). (G) Genome‐wide distribution of endogenous peptides in maize. The peptide‐rich regions on the right side of each chromosome are indicated by green circles. (H) Normalized distribution of different peptide expression categories along the chromosomal arms. AP2, apetala2; ARF, auxin response factors; BES1, bri1 ems suppressor 1; bHLH, basic helix‐loop‐helix; bZIP, basic leucine zipper; B3, b3 domain containing transcription factor; chr, chromosome; C2H2, cys2/his2; C3H, cysteine 3 histidine; Dof, dna binding with one finger; E2F/DP, eukaryotic 2 transcription factor/dimerization partner; EIL, ethylene insensitive3‐like; Em20DAP, embryo 20 DAP; GP, germinated pollen; En8DAP, endosperm 8 days after pollination; EP, ear primordium 1 mm; ERF, ethylene responsive factor; FS, female spikelet; GRAS, gibberellin‐acid insensitive (gai)/repressor of ga1 (rga)/scarecrow (scr); GeBP, glabrous‐enhancer‐binding protein; GRF, growth regulating factor; G2‐like, golden2‐like; HD‐ZIP, homeodomain‐leucine zipper; HSF, heat shock transcription factor; IND, 6–7 internode; JLB3, juvenile leaf blade 3; LBD, lateral organ boundaries domain; MIKC, mads intervening keratin‐like c‐terminal domain; ML8, mature leaf 8; MYB, myeloblastosis; NAC, no apical meristem/ataf1/2/cup‐shaped cotyledon; NF‐YA, nuclear transcription factor y subunit alpha; NIN‐like, nodule inception‐like; PR5D, primary root 5 days; RAV, related to abi3/vp1; SBP, squamosa promoter binding protein; SiU, unpollinated silk; SR7D, secondary root 7 days; SRS, shi‐related sequence; TALE, three‐amino‐acid loop extension; Tas, tassel; TCP, teosinte branched1/cycloidea/proliferating cell factor; VM19D, vegetative meristem 19 days; WOX, wuschel‐related homeobox; ZF‐HD, zinc finger‐homeodomain.

In this study, based on a peptidogenomic strategy developed previously [14], we analyzed 13 maize tissues, including the 6–7 internode (IND), juvenile leaf blade 3 (JLB3), mature leaf 8 (ML8), primary root 5 days (PR5D), secondary root 7 days (SR7D), vegetative meristem 19 days (VM19D), tassel (Tas), germinated pollen (GP), unpollinated silk (SiU), female spikelet (FS), ear primordium 1 mm (EP), endosperm 8 days after pollination (En8DAP), and embryo 20 DAP (Em20DAP) to generate a comprehensive and robust peptide atlas (Figures 1A, S1A,B and Table S1). Using a three‐frame translation of canonical coding sequences (CDSs), we identified 6100 nonredundant endogenous peptides originating from 3692 unique genes (Table S2). A significant portion (90.95%) of the peptidome was derived from in‐frame CDSs, and only 9.05% of the peptides came from out‐of‐frame CDSs. While in‐frame peptides are well‐known molecular players, the identification of out‐of‐frame peptides in our study signifies an emerging study domain suggesting that both types of peptides could be essential for a comprehensive understanding of biological processes in plants. Among the vegetative tissues, SR7D contributed the highest number of peptides (2717), whereas En8DAP had the highest count (2899) among reproductive tissues (Figure 1A). In addition, approximately 94% (5748) of the peptides were shorter than 23 amino acids, with an average molecular weight of 1472.97 Da; moreover, 96.4% (5881) had a molecular weight less than 2500 Da (Figure S2A,B).

### Dynamics of maize peptidome across growth transitions

The maize peptidome showed variation in expression across developmental stages. On the basis of their expression specificity, the endogenous peptides were categorized as tissue‐specific, tissue‐enhanced, and mixed (see Supporting Information Methods) (Figure S3). Approximately 21–680 tissue‐specific, 2–877 tissue‐enhanced, and 19–1000 mixed peptides were identified per tissue (Figures 1B, S1C,D and Table S2). The peptidome of SR7D contained the highest peptide count across these categories, followed by En8DAP. The endogenous peptides displayed diverse expression patterns spanning the vegetative and reproductive phases, highlighting the complexity and dynamic nature of maize peptidome (Figure 1C). Furthermore, the tissue‐enhanced and mixed peptides demonstrated more dynamic expression across both vegetative and reproductive phases compared with tissue‐specific peptides (Figure 1D,E).

Traditional overenrichment analysis of 260 endogenous peptides derived from 36 transcription factors (TFs) revealed that peptides derived from NAC (no apical meristem, ATAF1/2, cup‐shaped cotyledon) TFs, WRKY TFs, and Golden2‐like TFs were overrepresented in vegetative tissues, including the mature leaf, and vegetative meristem (Figures 1F and S4). On the other hand, peptides originating from basic helix‐loop‐helix (bHLH), APETALAS (AP2), and Whirly TFs were overrepresented in reproductive tissues, such as the fertilized embryo, GP, and SiU, respectively. These peptides may regulate TF functions by interacting with other proteins or bind directly to specific functional motifs such as activation domain or DNA‐binding domain of TFs to modulate tissue‐specific processes such as soil nutrient uptake by roots, and photosynthesis and stomatal regulation in the leaf.

### Distribution of peptide expression categories across maize chromosomes

The maize chromosomes demonstrated a wide range of peptide‐encoding capabilities, with chromosome 1 encoding the highest percentage (24.57%) (Figure S2C). All peptide expression categories were unevenly distributed across the chromosomes. A relatively higher percentage, that is, 30.57% (727) of tissue‐enhanced, 29.74% (423) of mixed, and 29.04% (668) of tissue‐specific peptides, was observed near the telomeric regions (Figure 1H). A total of 49 peptide‐rich regions (defined by 6‐Mb windows) encompassing 2403 (39.39%) peptides were identified, with most of these regions located near the telomeres (Figure 1G). These peptides may interact with the ribonucleoprotein enzyme complex, the telomerase in maintaining the telomere length to prevent the loss of terminal chromosomal DNA during cell division in maize. The distance between two adjacent peptides was used to determine the extent of peptide coverage across the genome. In this study, 59.17% (1361), 61.01% (1451), and 46.76% (665) of the adjacent tissue‐specific, tissue‐enhanced, and mixed peptides, respectively, were found less than 500 kb apart (Figure S2D,E).

Next, we compared the location of the expression specificity categories with their respective gene models. Interestingly, 89.73% (2064), 90.37% (2149), and 90.71% (1290) of the tissue‐specific, tissue‐enhanced, and mixed peptides, respectively, were located within 2 kb of the canonical translation start site (TSS) (Figure S2F–H). Furthermore, analysis of the nucleotide sequence of the peptide–source transcripts revealed a preponderance of non‐AUG TSSs (97.57%) which is consistent with previous peptidomic research that over 90% of naturally occurring peptides initiate with a non‐AUG start codon [14].

### Weighted gene coexpression network analysis (WGCNA) of peptidome and proteome

**Figure 2:**
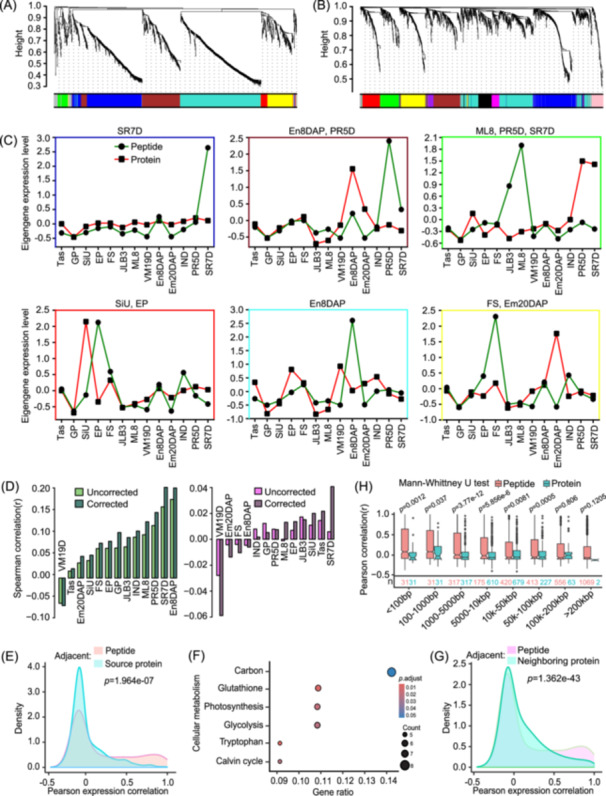
WGCNA and genome‐wide coexpression of endogenous peptides with associated proteins. (A) Coexpression dendrograms and the corresponding modules generated for the peptide network. Each module, represented by colored boxes, contains peptides with similar expression profiles within the network. (B) Coexpression dendrograms and the corresponding modules generated for source proteins network. Each module, represented by colored boxes, contains proteins with similar expression profiles within the protein coexpression network. (C) Eigengene profiles of peptide (green) and source protein (red) modules derived from WGCNA. Each module is represented by a different panel. The border of each plot is manually annotated with a different color corresponding to the module representing the tissue in which it shows the highest expression. (D) Tissuewise Spearman correlation between peptides versus source proteins (left) and peptides versus neighboring proteins (right). (E) Density distributions of Pearson's *r* between adjacent peptide–peptide pairs and peptide–source protein pairs. (F) Enrichment of biological processes for source proteins showing higher expression correlation (*r* > 0.70) with endogenous peptides. (G) Density distribution of Pearson's *r* between adjacent peptide–peptide pairs and peptide neighboring protein pairs. (H) Pearson's *r* distribution for peptide–peptide, and neighboring peptide–protein pairs at different distance intervals. Em20DAP, embryo 20 DAP; GP, germinated pollen; En8DAP, endosperm 8 days after pollination; EP, ear primordium 1 mm; FS, female spikelet; IND, 6–7 internode; JLB3, juvenile leaf blade 3; ML8, mature leaf 8; PR5D, primary root 5 days; SiU, unpollinated silk; SR7D, secondary root 7 days; Tas, tassel; VM19D, vegetative meristem 19 days; WGCNA, weighted gene coexpression network analysis.

The WGCNA R package was used to group similarly expressed peptides or peptides‐source proteins across maize development into modules (clusters) (Figure 2A,B). A soft threshold of 5 with a scale‐free topology fit > 0.80 and mean connectivity below 100 was deemed satisfactory for both coexpression networks (Figure S5A,B). Using this approach, we grouped 1467 peptides into six modules and 1690 peptide–source proteins into 10 distinct coexpression modules (Figures 2C, S6, and Tables S3, S4). Subsequently, the eigengene profile of each module was plotted to identify the tissue in which each module showed the highest expression. The peptide modules' eigengenes exhibited different abundance patterns compared with the protein modules' eigengenes throughout maize development (Figure 2C) suggesting that peptides may perform distinct biological functions from their source proteins, such as acting as signaling molecules or regulating other proteins. Their expression patterns may therefore reflect these unique roles, which are modulated according to developmental stages. Furthermore, the peptidome may operate through distinct regulatory mechanisms including posttranslational modifications like proteolytic cleavage, phosphorylation, ubiquitination, and alternative splicing enabling peptides to have specialized roles in developmental processes.

### Interaction relationship analysis of peptides and source protein modules

Both peptides and their source proteins within the modules showed weaker interactions than peptides and source proteins from different modules, suggesting a low degree of intermodular interdependence (Figure S7A,C). Clustering of the module eigengenes (ME) by expression correlation with maize tissues revealed two main clusters for each data set (Figure S8A,B). The ME of the green, blue, and brown peptide modules was weakly positively correlated with vegetative tissues, including ML8 (*r* (average) = 0.50), SR7D (*r* (average) = 0.54), and PR5D (*r* (average) = 0.53), respectively (Figure S7B). The turquoise, red, and yellow modules displayed weak positive correlations with reproductive tissues, including En8DAP (*r* (average) = 0.54), EP (*r* (average) = 0.46), and FS (*r* (average) = 0.49), respectively. However, the MEs of the same source proteins modules did not correlate well with maize tissues, except the red module (Figure S7D). The MEs of red, pink, and black modules displayed a weak positive correlation with reproductive tissues, including SiU, GP, and Tas, respectively.

Although the correlation between peptide modules and maize tissues was weak but more consistent than that of the source protein modules, this prompted us to identify several functional signatures for the peptide modules. For example, the blue module, corresponding to SR7D, contained peptides such as LGHLHTRTL, LTIDNQVST, and EPAAEATSREVAVEE derived from root hair defective 3 (RHD3) GTP‐binding protein [15], roothairless1 [16], and plasma membrane‐associated cation‐binding protein 1 (PCAP1) [17], respectively. These genes have been reported to regulate root hair growth [15, 16] and root hydrotropism [17]. Similarly, the green module, highly expressed in mature leaf, contained peptides derived from proteins involved in the light reactions of photosynthesis. The turquoise module, specific to En8DAP, included a peptide (P92: LQESLGGNAKLAM) derived from a class of motor proteins known as kinesins. Members of the kinesin protein family have been implicated in endosperm development in maize [18, 19]. Peptide–protein interaction analysis may reveal how specific peptidomes contribute to regulate developmental stages and physiological processes, thus providing a deeper understanding of the modular functions in maize development.

### Peptide to protein coexpression correlation at genomic scale

Studies in *Arabidopsis* and maize have documented a weak positive correlation between the transcriptome and proteome [9, 10]. However, the extent of correlation between the peptidome and proteome remains unknown. In this study, the Spearman correlation between peptides and their source proteins ranged from −0.06 to 0.21, while it hovered around zero for peptides and the neighboring proteins within a tissue (Figure 2D and Table S5), implying that tissue‐specific regulatory events of peptides might be different from the events involving proteins, or peptides may function contrarily to proteins in the same regulatory event. Both the peptide and associated protein data sets exhibited similar patterns as revealed by principal component analysis (Figure S9A–C). However, the principal components of the peptide data set were distinct from their source and neighboring protein data sets (Figure S9D,E).

The extent of coexpression correlation between peptides and their associated proteins was assessed at the genomic scale. We observed significantly negative to weak positive coexpression correlations (*r* > − 0.46 and < 0.70) for 95.95% of the peptide–source protein pairs (*p* = 1.694 × 10^−7^ two‐sided Wilcoxon signed‐rank test) (Figure 2E). Only 269 peptide–source protein pairs exhibited a higher coexpression correlation (*r* > 0.70), and their source proteins were mainly enriched in cellular metabolism, including the primordial glycolysis pathway (Figure 2F). In contrast, only 35 peptide–neighboring pairs in the data set showed significantly higher coexpression correlation (*r* > 0.70) as indicated by the two‐sided Wilcoxon signed‐rank test (*p* = 1.362 × 10^−43^) (Figure 2G). Furthermore, the expression correlation was inversely proportional to the distance (up to 200 kb) for peptide–peptide pairs (Figure 2H). The remarkably weaker peptide‐to‐protein correlations and the significant nonconservation of peptides coexpression with their associated proteins may be attributed to several factors: the movement of peptides and proteins from the tissues where they are synthesized, alternative splicing, stability, posttranslational modifications, and the dynamic nature of biological interactions between peptides and proteins in the cell.

## CONCLUSION

In summary, this study generated a comprehensive and reliable maize peptide atlas, offering novel insights into the dynamic expression patterns and tissue‐specific distribution of endogenous peptides across developmental stages. This resource presents new opportunities for understanding peptide‐based regulatory mechanisms in maize. The peptidome displayed a relatively stronger correlation with maize tissues compared with their source protein counterparts. Notably, the genome‐wide coexpression analysis revealed a significant lack of conservation between the peptidome and proteome, underscoring the unique regulatory mechanisms governing peptide function, independent of protein expression. Furthermore, this atlas serves as a valuable resource for future research, as it can be integrated with other omics data, such as transcriptomics and proteomics, to enable a more holistic understanding of gene regulation and function in plants.

## AUTHOR CONTRIBUTIONS

**Usman Ali**: Writing—original draft; investigation; writing—review and editing. **Lei Tian**: Investigation; formal analysis. **Ruihong Tang**: Investigation. **Shunxi Wang**: Writing—review and editing; validation. **Weiwei Luo**: Validation; writing—review and editing. **Shanshan Liu**: Investigation. **Jinghua Zhang**: Investigation. **Liuji Wu**: Conceptualization; writing—review and editing; funding acquisition; supervision; project administration.

## CONFLICT OF INTEREST STATEMENT

The authors declare no conflict of interest.

## ETHICS STATEMENT

No animals or human participants were involved in this study.

## Supporting information


**Figure S1:** Data reproducibility and endogenous peptide distribution in maize tissues.
**Figure S2:** Characteristics and genome‐wide distribution of maize endogenous peptides.
**Figure S3:** Peptide expression specificity categories in maize.
**Figure S4:** Hierarchical clustering of peptide abundance (expression) levels derived from transcription factors (TFs).
**Figure S5:** Assessment of optimal soft threshold powers used in WGCNA to construct peptide and source protein coexpression networks.
**Figure S6:** Eigengene profiles of protein modules derived from WGCNA.
**Figure S7:** Interaction relationships of peptide and source protein modules.
**Figure S8:** Hierarchical clustering and heatmap of the adjacencies in peptide and source protein module eigengenes.
**Figure S9:** Comparison of PCA between peptides and peptide‐associated proteins.

**Table S1:** Pearson correlations of biological replicates within each tissue.
**Table S2:** List of non‐redundant endogenous peptides identified in maize using the peptidogenomics pipeline.
**Table S3:** WGCNA‐derived peptide modules. Modules highly expressed in specific maize tissues are shown in Figure 2.
**Table S4:** WGCNA‐derived protein modules. Modules highly expressed in specific maize tissues of maize are shown in Figure 2 and Figure S6.
**Table S5:** Tissue‐wise Pearson and Spearman correlations between endogenous peptides and their associated proteins.

## Data Availability

The data that supports the findings of this study are available in the supplementary material of this article. All the mass spectrometry data have been deposited in the ProteomeXchange Consortium via the PRIDE partner repository with data set identifiers PXD055863 (https://www.ebi.ac.uk/pride/profile/reviewer_pxd055863). The data and scripts used are saved in GitHub (https://github.com/WljLab/Peptide-atlas-of-maize). Supplementary materials (methods, figures, tables, graphical abstract, slides, videos, Chinese translated version and update materials) may be found in the online DOI or iMeta Science http://www.imeta.science/.
