# Supplementary material for: A comprehensive atlas of endogenous peptides in maize
Source: Imeta. 2024 Nov 11;3(6):e247. doi: 10.1002/imt2.247 (PMC11683474; doi:10.1002/imt2.247)
Supplement: Supplementary file 1 — Figure S1: Data reproducibility and endogenous peptide distribution in maize tissues. Figure S2: Characteristics and genome‐wide distribution of maize endogenous peptides. Figure S3: Peptide expression specificity categories in maize. Figure S4: Hierarchical clustering of peptide abundance (expression) levels derived from transcription factors (TFs). Figure S5: Assessment of optimal soft threshold powers used in WGCNA to construct peptide and source protein coexpression networks. Figure S6: Eigengene profiles of protein modules derived from WGCNA. Figure S7: Interaction relationships of peptide and source protein modules. Figure S8: Hierarchical clustering and heatmap of the adjacencies in peptide and source protein module eigengenes. Figure S9: Comparison of PCA between peptides and peptide‐associated proteins. [file IMT2-3-e247-s002.docx]

**Supplementing information to**

**A Comprehensive Atlas of Endogenous Peptides in Maize**

**Running title:** The peptide atlas of maize

Usman Ali^1#^, Lei Tian^1#^, Ruihong Tang^1^, Shunxi Wang^1^, Weiwei Luo^1^, Shanshan Liu^1^, Jinghua Zhang^1^, Liuji Wu^1^^*^

^1^National Key Laboratory of Wheat and Maize Crop Science, College of Agronomy, Henan Agricultural University, Zhengzhou, 450002, China.

^#^These authors contributed equally: Usman Ali, Lei Tian

^*^Correspondence: [wlj200120@163.com](mailto:wlj200120@163.com) (Liuji Wu)

**Supplementary materials and methods**

**Plant material**

In this study, the maize B73 inbred line was used for endogenous peptide profiling. Samples (~2 g) were collected from 13 tissues including vegetative meristem 19 days (VM19D), 6-7^th^ internode (IND), Juvenile Leaf Blade 3 (JLB3), Mature leaf 8 (ML8), primary root 5 days (PR5D), secondary root 7 days (SR7D), Tassel (Tas), germinated pollen (GP), unpollinated silk (SiU), ear primordium 1 mm (EP), female spikelet (FS), endosperm 8 DAP (En8DAP), and Embryo 20 DAP (Em20DAP). The samples were immediately frozen in liquid nitrogen and kept at -80 ℃ until further analysis.

**Peptide extraction and analysis by mass spectrometry**

Peptide extraction and mass spectrometry analysis was performed as described previously [1, 2]. The collected samples were ground into fine powder in liquid nitrogen followed by heating in water bath at 95 ℃ for 5 min. Then, precipitation of the samples was carried out in 10% (w/v) trichloroacetic acid/acetone solution at -20 ℃ for 1 h followed by washing with cold acetone to obtain a colorless supernatant which was then discarded. The precipitate was vacuum-dried and subsequently resuspended in a solution containing 1% TFA and plant protease inhibitor cocktail (Sigma, USA) followed by incubation at 4 ℃ for 1 h. Then ultrasonication of the fractions was carried out in ice followed by centrifugation at 10000 g at 4 ℃ for 20 min. A centrifuge filter (Millipore, MA, USA) of 10 kDa molecular-weight-cutoff was used to filter the supernatant. The peptide fractions after desalting using C18 cartridges (Empore, SPE Cartridges C18, 7 mm inner diameter, 3 ml volume; Sigma), were transferred to vacuum centrifugation concentrator for vacuum evaporation and finally resuspended in 40 ul of TFA solution (0.1%) for analysis by LC-MS/MS.

The Q Exactive mass spectrometer was used for the profiling of endogenous peptides [2]. The peptide mixtures (5 µg) were loaded onto C18 reversed-phase column (Thermo Scientific Easy Column) of 10 cm length, 75 µm inner diameter, and 3 µm resin in buffer A containing 2% acetonitrile and 0.1% formic acid. The peptide fractions were separated using a linear gradient of buffer B containing 80% acetonitrile and 0.1% formic acid. The separation process was carried out at a regulated flow rate of 250 nl/min utilizing IntelliFlow technology for a total duration of 120 min. The MS data acquisition was performed utilizing data-dependent top-10 method. This approach involved the dynamic selection of the most abundant precursor ions from the survey scan (300-1800 m/z) for fragmentation using higher energy collisional dissociation (HCD). The target value was determined using predictive automatic gain control. The duration of dynamic exclusion was 25 s. The resolution for survey scans was set to 70,000 at 200 m/z, whereas the resolution for HCD spectra was set at 17,500 at the same m/z. At 30 eV, the normalized collision energy was set, and at 0.1%, the underfill ratio was defined, indicating the lowest possible percentage of the target value that could be obtained at maximum fill time. The peptide recognition mode was activated at the time the instrument was operated.

**Identification of endogenous peptides by peptidogenomics strategy**

The putative peptide database was first constructed using 3-frame translation of canonical CDS, ensuring a more comprehensive identification of peptides by considering all possible reading frames [3], unlike proteomics methods that rely on known protein annotations [4, 5] with greater possibility of missing alternative products. At each stop codon, the peptide was terminated and the sequence of the subsequent peptide started at nucleotide next to the stop codon. The ambiguous nucleotides (denoted by “N”) or characters were substituted by random nucleotides. For each peptide, the genomic coordinates and orientation were recorded and the amino acid sequence of the putative peptides resulting from each chromosome was saved in FASTA format file. Each mass spectrometry data was matched with customized database and ensembl protein database (<ftp://ftp.ensemblgenomes.org/pub/plants/release-41/fasta/zea_mays>) by mascot software (6G database split into 15 small libraries, 15 non-enzyme digestion libraries). All peptides with Mascot score ≥ 25 were restored. The mass spectrometry data was searched against the identified peptides using maxquant software (FDR<0.05 parameter). All sample mass spectrometry data were checked in the same batch of the above peptide self-built library to extract peptide intensity values. The final list of peptides obtained from Ensembl protein and customized peptidogenomic databases after filtering (FDR cutoff (score ≥ 25; FDR < 0.05), were matched with the CDSs of their corresponding source genes followed by manual verification of their MS/MS spectra. Peptides with same sequences originating from different source genes were discarded.

**Genome-wide distribution of endogenous peptides**

We calculated the peptide density by analyzing the overlapping 6 Mb windows with a 3 Mb step size along the genome. Hotspot regions were determined as genomic segments spanning 6-Mb with a peptide count exceeding 40. The annotated maize genome was retrieved from <https://plants.ensembl.org/index.html> and the physical coordinates of TSSs were extracted. In order to create a frequency plot showing the distance between each peptide and its TSS, we searched for the TSS that was closest to each peptide. The position of peptides was then divided by the chromosomal arm length to determine the number of peptides encoded by each chromosome.

**Principal component analysis (PCA)**

The PCA was carried out according to the method previously described [4]. First the average of three biological replicates was calculated. The data were normalized using log2 calculation method and subjected to principal component analysis using stats package's R function prcomp () with default settings. Each principal component's (PC) proportion of variance was extracted using the summary () function applied to the prcomp objects. The first 4 PCs for each tissue were obtained by extracting the values from the "x" matrix of each prcomp object (prcomp$x[,1:4]). These data were then utilized to generate PCA graphs using the function, R plot (). Subsequently, by indexing the "rotation" matrix (prcomp$rotation[,1:4]), we were able to extract from the prcomp objects, the contribution of each peptide, peptide-source protein, and peptide-neighboring protein to each of the first four principal component analyses (PCAs) for the individual dataset. We then extracted the absolute values from the individual matrix. The function R plot () was then utilized to plot the peptide contributions against the source proteins and neighboring proteins contributions for all the 4 PCs. At final step we determined the Pearsons correlation coefficients for all these comparisons by applying the function cor () in R package. The protein abundance values as dNSAF from 13 maize tissues of maize were retrieved from a previous study [4].

**Tissue-wise peptide to protein Spearman correlation**

For tissue wise peptide-to-protein correlation, the protein abundance data represented as dNSAF for the 13 maize tissues was retrieved from a previous study [4]. In this way, we computed spearman correlation for 6100 peptides from our peptide-dataset with 3692 unique proteins from the reported study across the 13 tissues. By utilizing the biological data from the aforementioned set to analyze tissue-wise peptide-to-protein correlations, we calculated the corrected Spearman correlation coefficient for each sample as described previously [6].

**Pearson expression correlation and peptide expression specificity categories**

The sample-wise pearson expression correlation coefficient (r) was calculated for all tissues using R function cor (). The correlation analysis was performed on the dataset with 6100 peptides and displayed as heatmap. The peptides were further categorized based on their expression specificity as described previously with some modifications [7, 8]. The peptides that were expressed only in one tissue were classified as tissue-specific while, those detected in all tissues were considered as core peptides. The peptides whose abundance level was at least 15 times higher in a particular tissue than their average abundance in all other tissues were categorized as tissue-enhanced peptides. All the remaining peptides were considered as mixed peptides.

**KEGG pathway enrichment analysis**

The KEGG pathway enrichment analysis was performed using clusterProfiler in R package. The ggplot2 package installed in R package was then used for plotting as described previously [9].

**Weighted gene coexpression network analysis (WGCNA)**

We constructed two coexpression networks: peptide-coexpression network covering 6100 peptides, and source proteins coexpression network encompassing 3692 proteins. The analysis was performed in WGCNA R package [10]. Identical parameters were set for the construction of each coexpression network. Adjacency matrices were generated using the adjacency () function with the ‘type’ parameter set to ‘signed’, employing Spearman correlation. Subsequently, we built topographical overlap matrices (TOMs) by setting all parameters to default in the TOMsimilarity () function and the TOM scores served as edge weights. Then, using the function hclust () with (method=“average"), co-expression modules were built by hierarchical clustering of the TOM distance (1-TOM). Finally, the mergeCloseModules () function was employed to merge similar modules with a cutHeight value of 0.15.

**Hierarchical clustering of peptides**

MultiExperiment Viewer (MeV v4.8; http://www.tm4.org/mev/) software was used for hierarchical clustering of the identified 6100 peptides including those derived from transcription factors (TF). The results of hierarchical clustering were displayed as heatmap in MeV software after normalizing the rows using the "Normalize Gene/Row Vectors" modification [4].

**Peptides coexpression with associated proteins**

This analysis was performed for the peptide-peptide adjacent pairs, peptide-source protein pairs, and peptide-neighboring protein adjacent pairs. We used bedtools (v.2.25.0) closest to assign peptides to their nearest proteins [11]. Each nearest protein was then matched to its subsequent neighbor protein which was taken as control. The pearson correlations were determined for these pairs. Based on this analysis, we identified possible coexpressed peptide-neighboring protein pairs based on correlation coefficients > 0.70, and when the correlation between the neighboring protein and the protein adjacent to the neighboring protein was below 0.70. In addition, we conducted a Fisher Z-transformation and used the paired function to quantify the disparity between the correlation coefficients for the peptide and the control. The function "t.test" from the R package "psych" (version 1.8.4) was used to conduct two-tailed tests for independent samples [12]. The criterion for our potential peptide-neighboring protein couples was an adjusted P-value of ≤ 0.01.

**REFERENCES**

1. Wang, Shunxi, Lei Tian, Haijun Liu, Xiang Li, Jinghua Zhang, Xueyan Chen, *et al*. 2020. “Large-Scale Discovery of Non-conventional Peptides in Maize and Arabidopsis through an Integrated Peptidogenomic Pipeline.” *Molecular Plant* 13: 1078-1093. <https://doi.org/10.1016/j.molp.2020.05.012>

2. Wang, Shunxi, Zan Chen, Lei Tian, Yezhang Ding, Jun Zhang, Jinlong Zhou, *et al.* 2019. “Comparative proteomics combined with analyses of transgenic plants reveal ZmREM1.3 mediates maize resistance to southern corn rust.” *Plant Biotechnol Journal* 17: 2153-2168. <https://doi.org/10.1111/pbi.13129>

3. Saghatelian, Alan, and Juan Pablo Couso. 2015. “Discovery and characterization of smORF-encoded bioactive polypeptides.” *Nature Chemical Biology* 11: 909-916. <https://doi.org/10.1038/nchembio.1964>

4. Walley, Justin W., Ryan C. Sartor, Zhouxin Shen, Robert J. Schmitz, Kevin J. Wu, Mark A. Urich, *et al*. 2016. “Integration of omic networks in a developmental atlas of maize.” *Science* 353: 814-818. <https://doi.org/10.1126/science.aag1125>

5. Cong, Qian, Ivan Anishchenko, Sergey Ovchinnikov, and David Baker. 2019. “Protein interaction networks revealed by proteome coevolution.” *Science* 365:185-189. <https://doi.org/10.1126/science.aaw6718>

6. Csárdi, Gábor, Alexander Franks, David S. Choi, Edoardo M. Airoldi, and D. Allan Drummond. 2015. “Accounting for experimental noise reveals that mRNA levels, amplified by post-transcriptional processes, largely determine steady-state protein levels in yeast.” *PLoS Genetics* 1: e1005206. <https://doi.org/10.1371/journal.pgen.1005206>

7. Mergner, Julia, Martin Frejno, Markus List, Michael Papacek, Xia Chen, Ajeet Chaudhary, *et al*. 2020. “Mass-spectrometry-based draft of the Arabidopsis proteome.” *Nature* 579: 409-414. <https://doi.org/10.1038/s41586-020-2094-2>

8. Uhlén, Mathias, Björn M. Hallström, Cecilia Lindskog, Adil Mardinoglu, Fredrik Pontén, and Jens Nielsen. 2016. “Transcriptomics resources of human tissues and organs.” *Molecular Systems Biology* 12: 862. <https://doi.org/10.15252/msb.20155865>

9. Liu, Yongle, Changgen Li, Aokang Qin, Wenli Deng, Rongrong Chen, Hongyang Yu, *et al*. 2024. “Genome-wide identification and transcriptome profiling expression analysis of the U-box E3 ubiquitin ligase gene family related to abiotic stress in maize (*Zea mays L*.).” *BMC Genomics* 25: 132. <https://doi.org/10.1186/s12864-024-10040-8>

10. Langfelder, Peter, and Steve Horvath. 2008. “WGCNA: an R package for weighted correlation network analysis.” *BMC Bioinformatics* 9: 559. <https://doi.org/10.1186/1471-2105-9-559>

11. Quinlan, Aron R., and Ira M. Hall. 2010. “BEDTools: a flexible suite of utilities for comparing genomic features.” *Bioinformatics* 26: 841-842. <https://doi.org/10.1093/bioinformatics/btq033>

12. Revelle, William R. 2017. “psych: Procedures for personality and psychological research.” <https://cran.r-project.org/web/packages/psych/index.html>


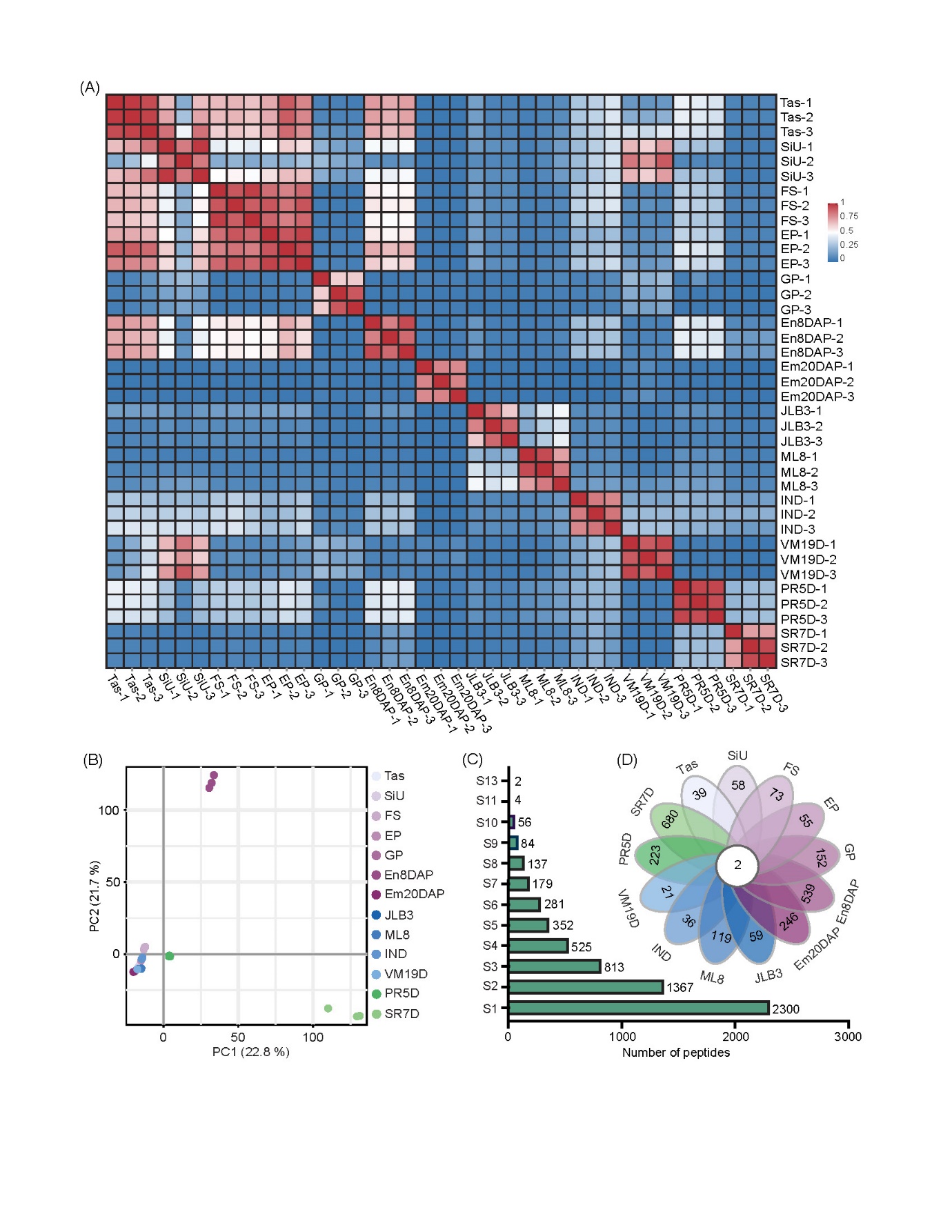


**Figure S1 Data reproducibility and endogenous peptides distribution in maize tissues.** (A) Sample-wise Pearson correlation of peptides within each tissue. (B) PCA analysis of all the samples. (C) Distribution of peptides identified in maize tissues. (D) Number of peptides identified in only one tissue.


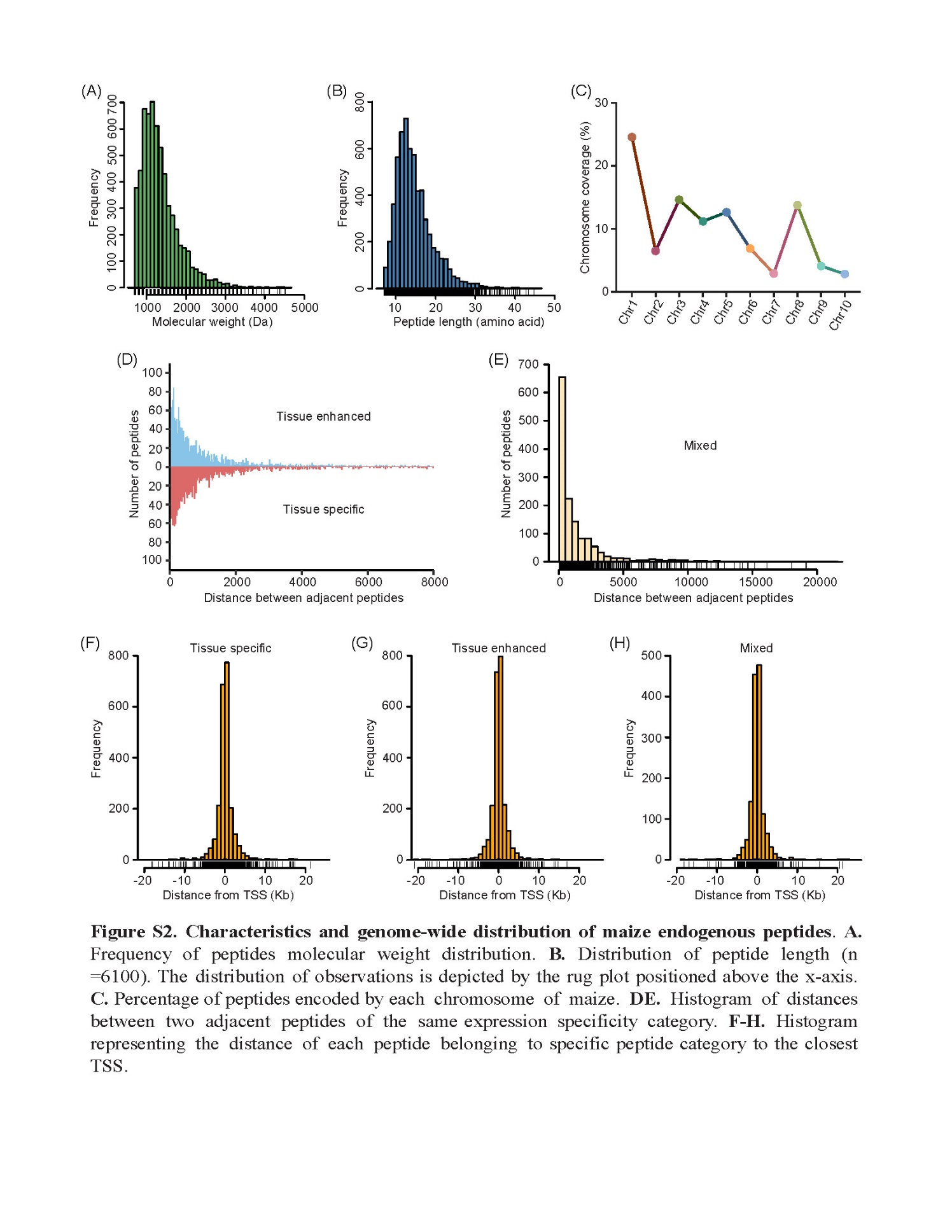


**Figure S2 Characteristics and genome-wide distribution of maize endogenous peptides.** (A) Frequency of peptides molecular weight distribution. (B) Distribution of peptide length (n = 6100). The distribution of observations is depicted by the rug plot positioned above the x-axis. (C) Percentage of peptides encoded by each chromosome of maize. (DE) Histogram of distances between two adjacent peptides of the same expression specificity category. (F-H) Histogram representing the distance of each peptide belonging to specific peptide category to the closest TSS.

**
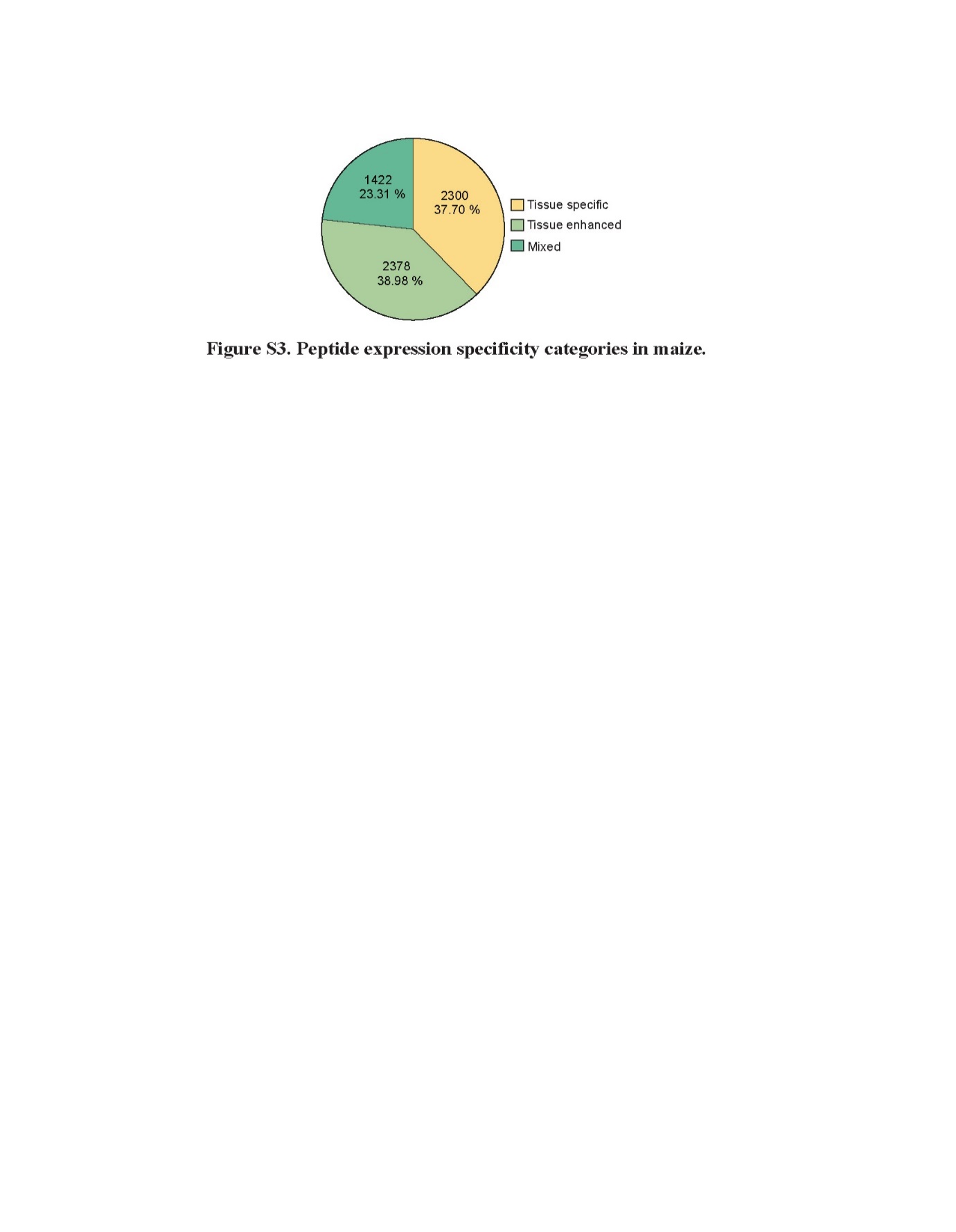
**

**Figure S3 Peptide expression specificity categories in maize.** The classification of peptides into different categories was based on their abundance pattern in maize tissues.


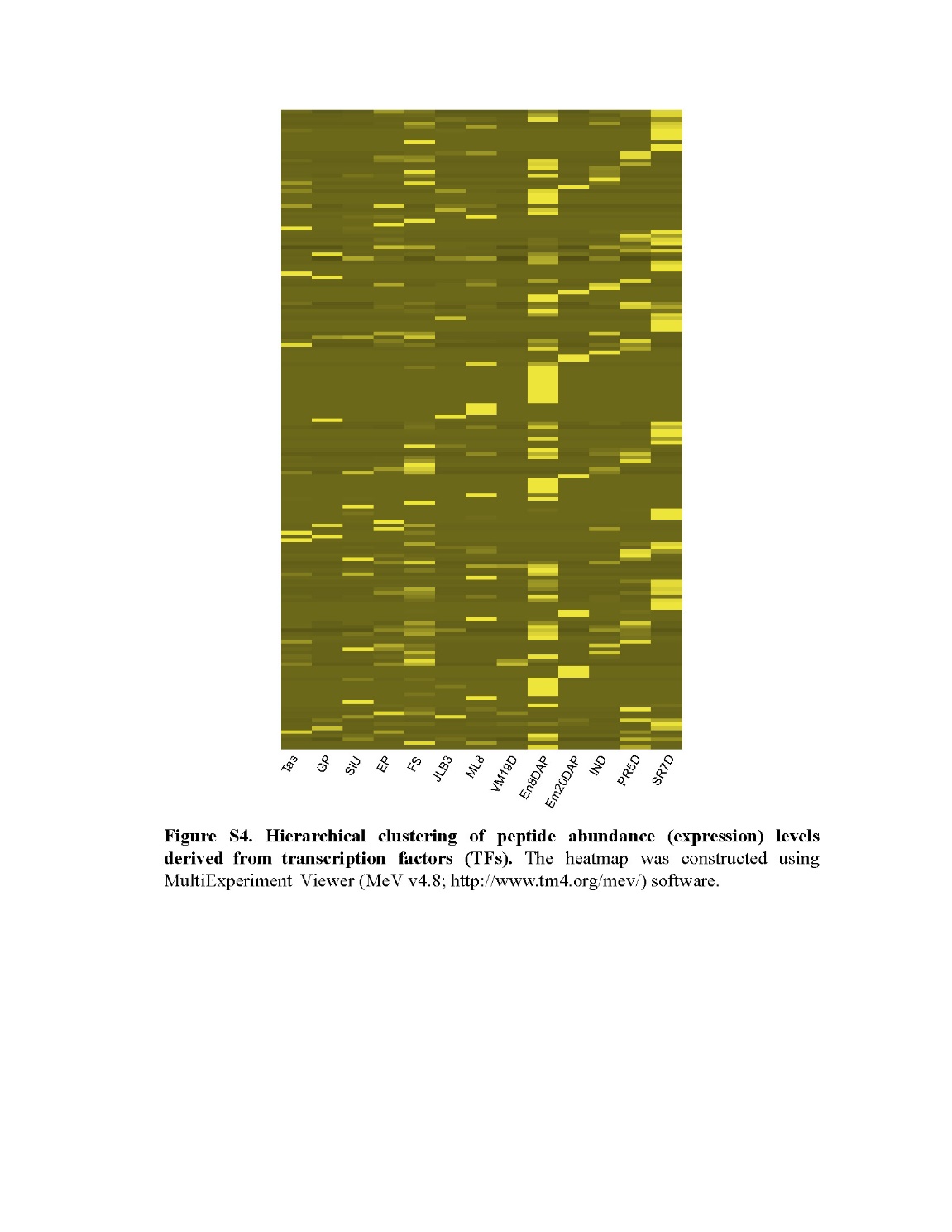


**Figure S4 Hierarchical clustering of peptide abundance (expression) levels derived from transcription factors (TFs).** The heatmap was constructed using MultiExperiment Viewer (MeV v4.8; http://www.tm4.org/mev/) software.


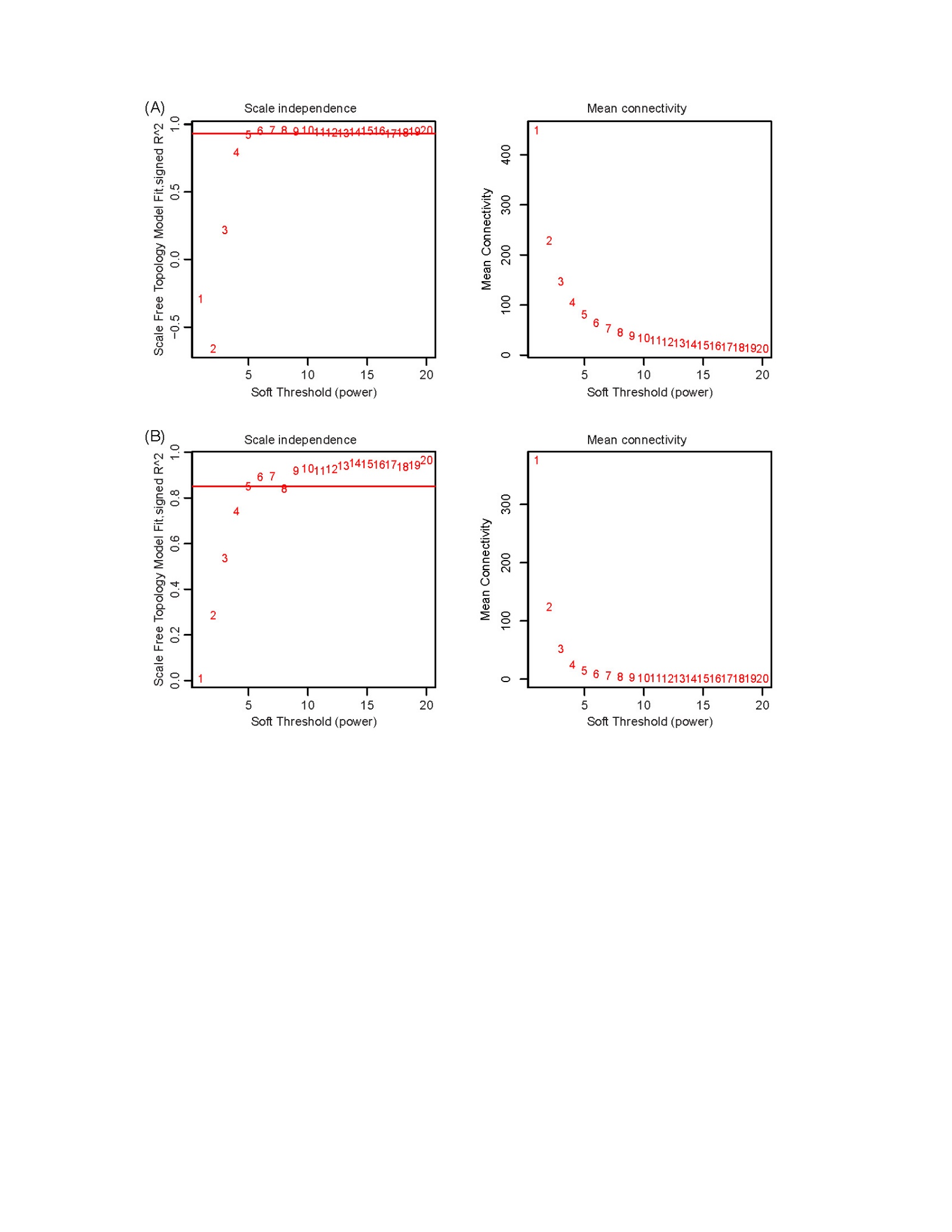


**Figure S5 Assessment of optimal soft threshold powers used in WGCNA to construct peptide and source protein coexpression networks.** (A) Range of soft threshold powers used in WGCNA to construct peptide coexpression networks based on Spearman expression correlation. The evaluation of soft threshold ranges was conducted by observing the scale independence (left) and mean connectivity in the peptide coexpression network (right). (B) Range of soft threshold powers used in WGCNA to construct protein coexpression networks based on Spearman expression correlation. The evaluation of soft threshold ranges was conducted by observing the scale independence (left) and mean connectivity in the protein coexpression network (right).


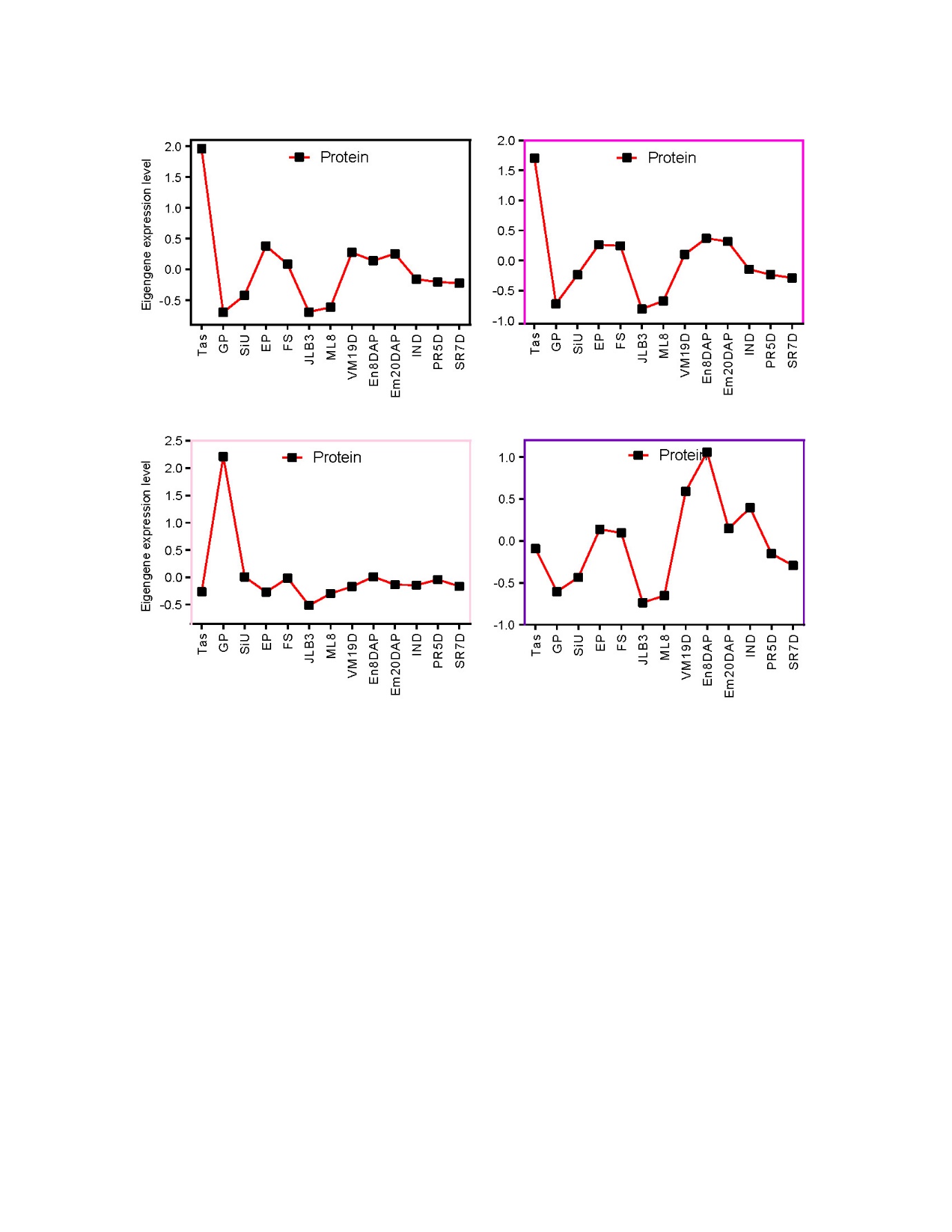


**Figure S6 Eigengene profiles of protein modules derived from WGCNA.** Each module is

represented by a different panel. The border of each plot is manually annotated with a different color corresponding to the module representing the tissue in which it shows the highest expression.


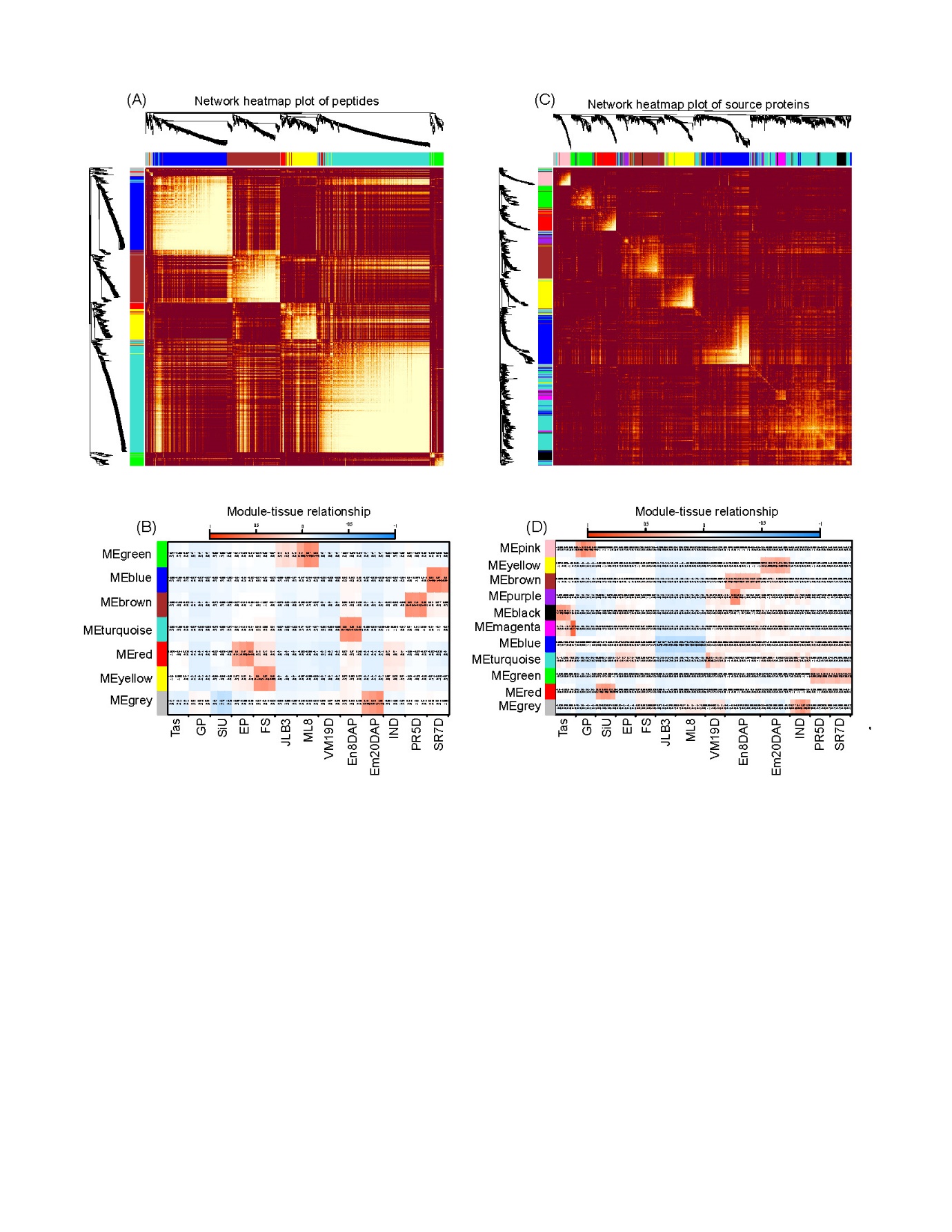


**Figure S7 Interaction relationships of peptide and source protein modules.** (A) Network heatmap of interaction relationship analysis of peptide modules. Different colors of horizontal and vertical axis represent different modules. The yellow color along the diagonal represents the degree of connectivity of the peptide modules. (B) Heatmap of the correlation between peptide module eigengenes and the maize tissues. (C) Network heatmap of interaction relationship analysis of source protein modules. Different colors of horizontal axis and vertical

axis represent different modules. The yellow color along the diagonal represents the degree of

connectivity of the source protein modules. (D) Heatmap of the correlation between source protein module eigengenes and the maize tissues.


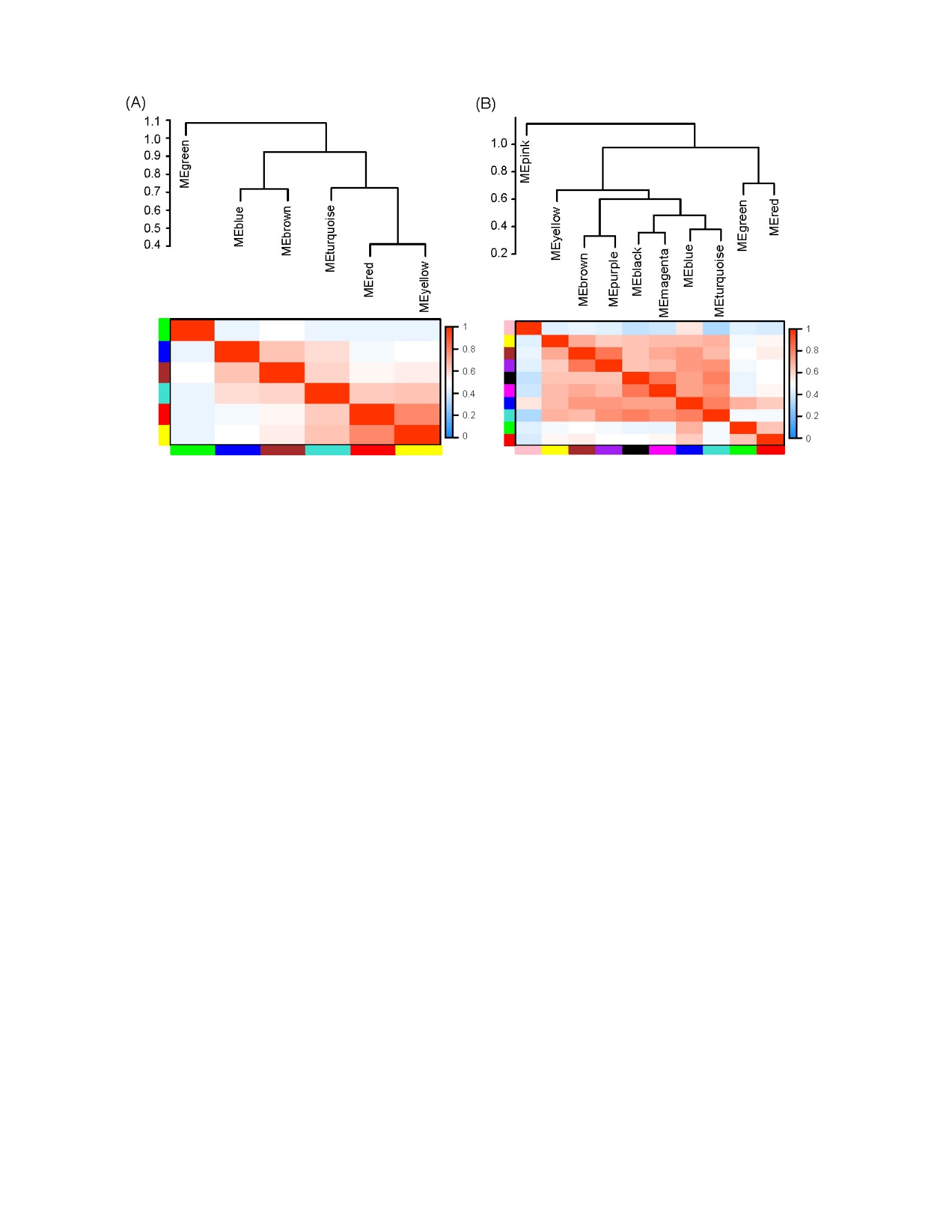


**Figure S8 Hierarchical clustering and heatmap of the adjacencies in peptide and source protein module eigengenes.** (A) Hierarchical clustering of the peptide modules eigengenes (top) and heatmap (bottom) of the adjacencies in the peptide network module eigengenes. (B) Hierarchical clustering of protein modules eigengenes (top) and heatmap (bottom) of the adjacencies in the protein network modules eigengenes.

**
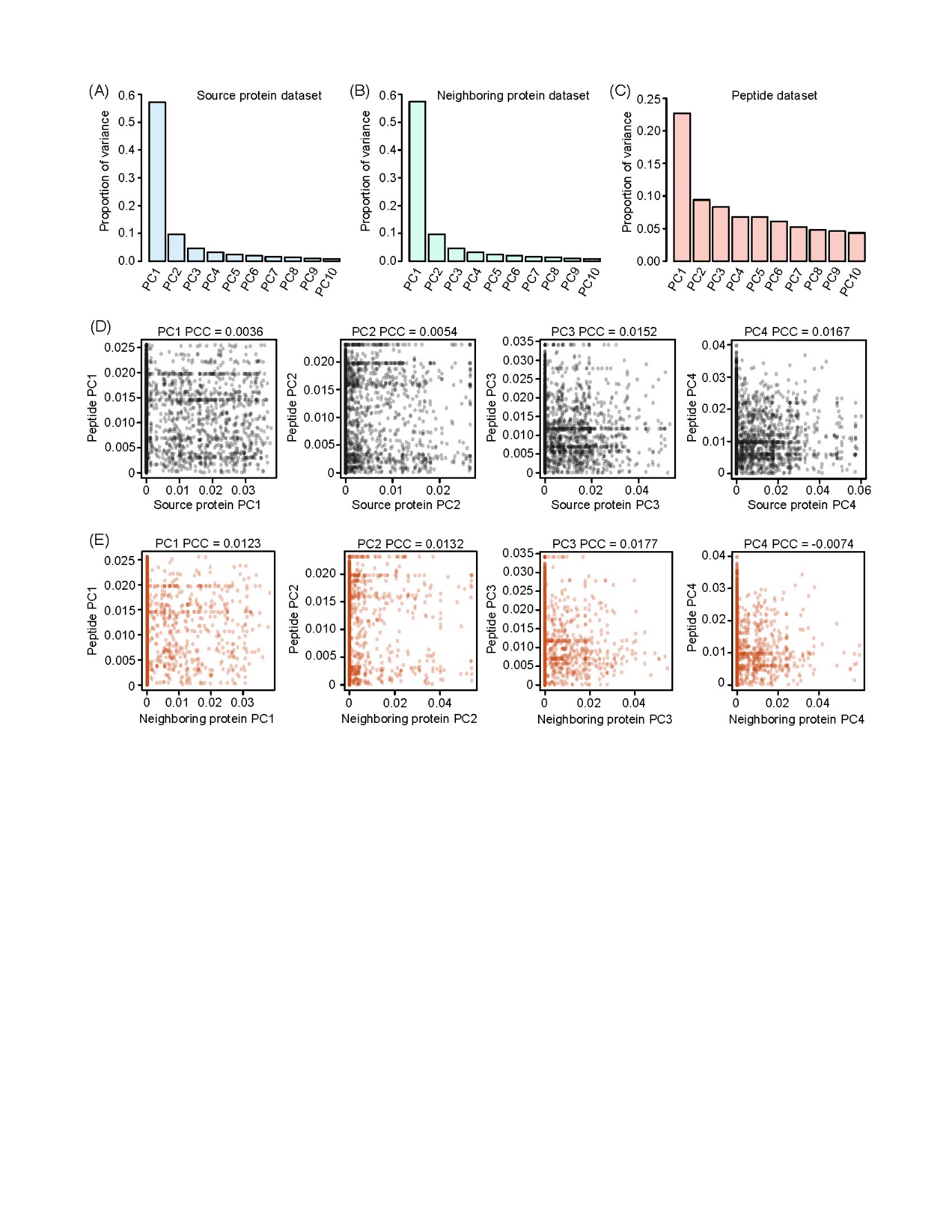
**

**Figure S9 Comparison of PCA between peptides and peptide-associated proteins.** (A) Contribution of each principal component to the total variance within the peptide dataset. (B) Contribution of each principal component to the total variance within the source protein dataset. (C) Contribution of each principal component to the total variance within the neighboring proteins dataset. (D) The contributions of each peptide from peptide dataset (y-axis) and source protein from source protein dataset are plotted against each other. The plots are displayed for the first four principal components (PCs). (E) The contributions of each peptide from peptide dataset (y-axis) and the neighboring protein from neighboring protein dataset are plotted against each other. The plots are displayed for the first four PCs.
